# Second Generation Antipsychotics Improve Sexual Dysfunction in Schizophrenia: A Randomised Controlled Trial

**DOI:** 10.1155/2011/596898

**Published:** 2011-01-16

**Authors:** Ahmed Mahmoud, Karen P. Hayhurst, Richard J. Drake, Shôn W. Lewis

**Affiliations:** ^1^Mental Health and Neurodegeneration Research Group, The University of Manchester, Manchester M13 9PL, UK; ^2^National Drug Evidence Centre (NDEC), School of Community Based Medicine, The University of Manchester, Manchester M15 6SZ, UK

## Abstract

The impact of antipsychotic drug treatment on sexual function was investigated during a randomised trial comparing first generation antipsychotics (FGAs) to (nonclozapine) second generation antipsychotics (SGAs). Sexual function and quality of life were (rater-blind) assessed in 42 patients with DSM-IV schizophrenia (aged 18–65) using the self-report version of the Derogatis Interview for Sexual Function (DISF-SR) and the Heinrichs Quality of Life Scale (QLS), prior to, and 12 weeks following, a change in medication from an FGA drug to either an FGA or SGA drug. SGAs significantly improved sexual function compared to FGAs. Change in sexual function was associated with change in quality of life. Where impaired sexual functioning is a distressing adverse effect of treatment with an FGA agent, consideration should be given to switching to an SGA.

## 1. Introduction

Sexual dysfunction is common in people with schizophrenia [1] affecting around a half of all patients [2–4] and likely to contribute to impaired quality of life [5–7]. Little systematic research has been carried out to date on the sexual behaviour of schizophrenic patients and a Cochrane review recently called for further research on the management of antipsychotic treatment-related sexual dysfunction [8]. 

Patients themselves report that the sexual side effects of medication are distressing [9]. For example, Finn and colleagues [10] found that patients with schizophrenia rated impotence, as a treatment side effect, as worse than any symptoms of the illness itself. Men may perceive sexual dysfunction as more distressing than women [11], but the experience of sexual dysfunction in women with schizophrenia has been underresearched. Sexual dysfunction can be a significant reason for nonadherence to prescribed antipsychotic treatment [1, 2, 12–15]. 

The relationship between antipsychotic drugs and sexual dysfunction is mediated in part by antipsychotic blockade of pituitary D2 receptors increasing prolactin secretion, although direct correlations have not been established between raised prolactin levels and clinical symptoms [16–18]. More recent studies highlight that a variety of mechanisms are likely to contribute to antipsychotic-related sexual dysfunction, including hyperprolactinaemia, sedation, and antagonism of a number of neurotransmitter receptors (*α*−adrenergic, dopaminergic, histaminic, and muscarinic) [19, 20]. 

Conventional, or first generation antipsychotic (FGA) drugs are associated with sexual side effects. Ghadirian and colleagues [21] found that up to 60% of men and 33% of women on FGAs reported changes in the quality of orgasm. A further study reported that chlorpromazine was associated with problems of orgasm and sulpiride with problems of arousal in female patients [3]. A Cochrane review highlights increased prolactin levels seen with sulpiride treatment [22]. Recent research demonstrates that as sulpiride is less lipophilic than other antipsychotics it has relatively high concentrations in the pituitary (outside the blood-brain barrier) compared to brain parenchyma [23]. This means that hyperprolactinaemia is more likely to occur at clinical doses of sulpiride. 

Individual second generation antipsychotic (SGA) drugs appear to be associated with less sexual dysfunction than that attributable to FGAs. One recent review concluded that risperidone had the largest impact on sexual dysfunction, followed by FGA drugs, then olanzapine, then clozapine, then quetiapine and, finally, aripiprazole [19]. Antipsychotic drugs with high D2-binding affinity have been identified as more likely to cause sexual dysfunction [1, 24] and risperidone may be associated with higher prolactin elevation than other SGAs [25–27]. Amisulpride is less lipophilic than risperidone or other SGAs and this may (as for sulpiride) explain why it is particularly identified with hyperprolactinaemia [26]. Bearing in mind the limitations associated with its design, data from the SOHO study, a large observational study funded by Lilly, indicates an advantage to both olanzapine and quetiapine, compared with risperidone and FGA drugs, across areas of sexual dysfunction [28–30]. A previous European study concluded that there were no real benefits of any of the SGAs over FGA treatment, apart from that seen with quetiapine in short-term trials [31]. Further studies have confirmed the association of both quetiapine [1, 32, 33] and aripiprazole [34, 35] with less severe sexual dysfunction. 

Sexual dysfunction is a difficult area to investigate. Sexual problems may be underreported to clinicians who, in turn, may underestimate the sexual side effects of antipsychotic treatment [1, 2, 6, 7, 13, 29, 31]. Researchers in the field have used a variety of assessment tools to measure sexual function, leading to difficulty when reviewing the published literature. 

We set out to examine sexual dysfunction in a subgroup of participants in a large trial. Patients already taking, but unresponsive to, an FGA were randomised to change to either another FGA or a non-clozapine SGA for 12 weeks while sexual function and quality of life were assessed.

We tested two hypotheses. Firstly, that sexual dysfunction contributes measurably to reduced health-related quality of life in patients with schizophrenia. Secondly, that sexual function and hence quality of life is better in patients with schizophrenia on SGAs than FGAs.

## 2. Materials and Methods

Patients (*N* = 42) were treated with an FGA drug prior to randomisation into the study. The study was conducted alongside CUtLASS (Cost Utility of the Latest Antipsychotics in Severe Schizophrenia [36–38]: a multicentre RCT that compared FGAs with SGA drugs. All participants of the substudy were recruited from one centre. The North West Multi-Centre Research Ethics Committee granted ethical approval with local approval obtained from each participating district.

### 2.1. Inclusion Criteria

Patients had a diagnosis of DSM-IV schizophrenia (and related disorders). The sample comprised patients recruited to CUtLASS (*N* = 20) and additional patients recruited from clinical practice (*N* = 22). All participants entered the study because of an identified need for a change to their FGA treatment, either because of ineffectiveness or intolerable side effects. No participants were referred because of sexual side effects. Patients were aged 18 to 65 years of both sexes. Patients receiving anticholinergic drugs were included.

### 2.2. Exclusion Criteria

Excluded patients included those with a diagnosis of schizoaffective disorder, patients with a comorbid history of alcohol or drug misuse within the last 12 months, and patients receiving treatment with more than one antipsychotic drug at baseline.

### 2.3. Randomisation

Randomisation, of all included patients, took place via a remote telephone service provided by the Medical Statistics Department at the Paterson Institute, Christie Hospital, Manchester, UK. The method of allocation was permuted blocks within strata with block sizes varying at random between four and 12. Arrangements were in place to ensure that the patient commenced their new antipsychotic drug treatment within hours of randomisation to either an FGA or an SGA drug. All subjects had a change to their antipsychotic drug and all completed the 12-week period on their assigned treatment. Randomised treatment allocation was revealed to the responsible clinician, the CUtLASS trial manager and the participant's GP.

### 2.4. Measures

Primary and secondary outcome measures used, respectively, were the self-report Derogatis Interview for Sexual Function (DISF-SR) [39] and the (rater-blinded) Quality of Life Scale (QLS) [40]. The Derogatis interview is a widely used, brief, self report, multidimensional and gender-keyed instrument designed to measure quality of current sexual functioning across five primary domains: sexual cognition and fantasy (5 items); sexual arousal (5 items); sexual behaviour and experiences (5 items); orgasm (6 items); sexual drive and relationship (4 items). The first three of these key domains are scored using a nine-point scale (from “0” as “not at all” through to “8” as “4 or more times per day”); orgasm is scored using a five-point scale (from “0” as “not at all” to “4” as “extremely”); the fifth domain, sexual drive, and relationship, is scored using a combination of nine- and five-point scales. The aggregate total score can be used repeatedly throughout efficacy or effectiveness studies without any significant practice effects or loss of validity [39].

The QLS [40] is the most widely used quality of life scale in the evaluation of psychopharmacological treatments for schizophrenia [41]. The 21-item scale covers four domains: social relationships; instrumental role functioning; intrapsychic foundations; activities of daily life. The QLS has been shown to be both sensitive to change and of clinical relevance [42].

### 2.5. Statistical Analysis and Power

Results were analysed using SPSS version 15 to carry out analyses of covariance (ANCOVAs) and correlational analyses (Pearson's *r* and Spearman's rho). An *a priori* power calculation suggested that the sample size obtained would have 90% power to detect a difference of 30 points on the DISF-SR between the two treatment groups, which was selected as a difference that both appeared clinically relevant and related to previous findings of differences between treatment groups [43].

## 3. Results

Table 1 shows demographic characteristics of the two treatment groups. Thirty-one men and 11 women with a mean age of 41 years (range 18 to 57 years) were randomised. 

The two groups were statistically comparable in terms of their ethnicity and age. The FGA arm had a lower median duration of illness and a higher proportion of males, compared with the SGA arm, although neither measure differed significantly between the two groups. 

Table 2 shows antipsychotic drug treatment received by the two groups.

Table 3 shows sexual function scores of the two treatment groups over the two assessment time points. 

Mean baseline total and subsection scores on the DISF-SR differed between the two groups (Table 3), but not significantly. Table 3 also shows that mean DISF-SR scores of the FGA-treated group worsened somewhat between the two time points.

There was no significant correlation between DISF-SR total score and QLS total score at baseline (*r* = 0.167, *P* = .290) and a weak (nonsignificant) correlation at week 12 (*r* = 0.293, *P* = .063). The trend relationship seen by week 12 of the study resulted from a significant correlation between score on QLS subscale 1 (interpersonal relations & social network) and score on Section 3 of the DISF-SR (sexual behaviour & experience: Spearman's rho = 0.356, *P* = .022). There was a significant correlation between change in DISF-SR total score and change in QLS total score from baseline to week 12 (Spearman's rho = 0.627, *P* < .001).

Twelve week DISF-SR total was modelled with an analysis of covariance. Final sexual function was significantly better (*F* = 7.45, *P* = .010) for patients randomised to an SGA (*N* = 20), compared to patients receiving an FGA (*N* = 22), covarying for baseline score and gender. Adjusted 12-week marginal mean DISF-SR score was 58.0 in the SGA group and 37.7 in the FGA group (difference 20.3, 95% confidence interval 5.2, 35.4). Age was dropped as a covariate as it was not independently significant. There was no significant difference between groups in final QLS total (*F* = 0.421, *P* = .521) or QLS subscales, covarying for gender and baseline score.

## 4. Discussion

Regarding the first hypothesis, sexual dysfunction did not have a straightforward relationship with reduced quality of life. It was unrelated at baseline but improvement in sexual function had a weak to moderate relationship with improving quality of life. This drove a trend-level association at final assessment. It may be that changes in sexual function have a more direct effect on quality of life than static sexual function, perhaps because over time there is a degree of adjustment to dysfunction. It is also worth noting that the QLS is an objective measure of quality of life, influenced by social function as well as participants' subjective experience; subjective quality of life may be more strongly related. 

Previous studies have linked male sexual dysfunction with poor quality of life in schizophrenia [6]. Although our results do not confirm this, again, the QLS may not be the best type of measure to detect a relationship. Total score on the QLS may not have sufficient sensitivity to detect sexual functioning-related changes in quality of life, and there is an argument for examining subscales of the QLS and DISF-SR. 

In terms of the second of our hypotheses, those who were in the SGA group had significantly better sexual function by the end of the trial. This suggests that switching from an FGA to an SGA can improve sexual function significantly. Note that none of the participants were referred into the study because of the sexual side effects of antipsychotic drug treatment. Sexual side effects can lessen with time following a treatment switch [44]. This reduction over time could, in part, explain the improvement in sexual function seen in the SGA group but fails to account for the deterioration seen in the FGA group. 

The study had a number of limitations. As stated earlier, the nature of the QLS, being determined largely by the deficit symptoms of schizophrenia [45], may explain the lack of effect of drug type on measured quality of life. Other treatment-related side effects, such as parkinsonism or sedation, which may also affect sexual function, were not measured in the study. The relatively small sample size did not permit an analysis of individual drugs. At the time of the study, some SGA drugs, such as ziprasidone and paliperidone, were not available in the UK, whilst others, such as zotepine, were little used. Female patients made up a relatively small proportion of those recruited limiting the generalisability of the findings. The study did, however, have the advantage of being a prospective, randomised comparison, one of a few to assess sexual function comprehensively. The self-report Derogatis scale was selected for this study because of its high validity and reliability. 

These findings add to the evidence that SGA drugs, as a group, have less adverse sexual side effects than those associated with FGA treatment. As an RCT of a change in medication it confirms the implication of existing nonrandomised survey data that switching antipsychotics can reduce the risk of treatment-associated sexual dysfunction [12, 19, 25]. Of course, it is not clear what effect switching within SGAs or from SGA to FGA would be, so it is uncertain whether this is an effect of switching class.

## 5. Conclusions

Switching from FGAs to SGAs improved sexual function compared to switching within FGAs. The relationship between the self-report measure of sexual function and the assessor-rated, objective quality of life measure used was complex, but it appeared that change in sexual function was associated with change in quality of life. Where impaired sexual functioning is a distressing adverse effect of treatment with an FGA agent consideration should be given to switching to an SGA.

## Figures and Tables

**Table 1:** Demographic Characteristics.

	FGA Arm (*N* = 22)	SGA Arm (*N* = 20)	*P*	test
Age				
Mean/SD	38.4 (10.0)	43.0 (11.7)	.173	*t* test
Median/Range	39.6/18–53	45.9/19–57		
Illness duration				
Mean/SD	14.4 (8.7)	18.6 (11.9)		
Median/Range	13.0/0.5–34	24.6/1–33	.182	Mann-Whitney
Gender				
* Male (%)*	19 (86%)	12 (60%)	.052	Chi Square
Ethnicity				
* White (%)*	20 (91%)	18 (90%)	1.000	Chi Square

**Table 2:** This table shows antipsychotic drug treatment received by the two groups.

	FGA Arm (*N* = 22)			SGA Arm (*N* = 20)	
*N*	Drug	Dose range	*N*	Drug	Dose range
2	Flupentixol	3–6 mg	2	Amisulpride	400 mg
1	Haloperidol	3 mg	7	Olanzapine	5–15 mg
3	Sulpiride	400–2000 mg	7	Quetiapine	100–700 mg
1	Trifluoperazine	15 mg	4	Risperidone	3–4 mg
7	Zuclopenthixol	4–20 mg			
1	Flupentixol depot	40 2/52			
1	Fluphenazine depot	25 2/52			
1	Pipotiazine palm.	50 3/52			
5	Zuclopenthixol depot	200 4/52–400 2/52			

**Table 3:** DISF-SR score.

DISF-SR Mean (SD)	FGA Group (*N* = 22)		SGA Group (*N* = 20)		
	Baseline	Week 12	Baseline	Week 12	*Baseline comparison*
Section 1: sexual cognition/fantasy	15.5 (12.7)	13.8 (10.6)	10.4 (9.3)	16.2 (11.0)	*P* = .130^†^
Section 2: sexual arousal	10.2 (8.2)	9.3 (8.9)	7.0 (8.4)	9.5 (9.0)	*P* = .202^†^
Section 3: sexual behaviour/experience	6.7 (5.8)	6.4 (5.9)	4.6 (6.8)	6.8 (8.5)	*P* = .085^‡^
Section 4: orgasm	9.1 (7.2)	8.0 (6.8)	5.6 (8.0)	9.0 (8.0)	*P* = .128^†^
Section 5: sexual drive	10.6 (4.5)	9.6 (5.0)	9.0 (4.6)	11.1 (5.0)	*P* = .236^†^
Total Score	52.6 (32.7)	47.5 (32.3)	36.4 (33.7)	52.6 (37.3)	*P* = .121^†^

^†^ *t*-test.

^‡^Mann-Whitney.

**NB: **A higher score on the DISF-SR scale is indicative of better sexual functioning.
